# Using EfficientNet-B7 (CNN), Variational Auto Encoder (VAE) and Siamese Twins’ Networks to Evaluate Human Exercises as Super Objects in a TSSCI Images

**DOI:** 10.3390/jpm13050874

**Published:** 2023-05-22

**Authors:** Yoram Segal, Ofer Hadar, Lenka Lhotska

**Affiliations:** 1 School of Electrical and Computer Engineering, Ben Gurion University of the Negev, Be’er-Sheva 84105001, Israel; hadar@bgu.ac.il; 2 Czech Institute of Informatics, Robotics and Cybernetics, Faculty of Biomedical Engineering, Czech Technical University in Prague, 160 00 Prague, Czech Republic; lenka.lhotska@cvut.cz

**Keywords:** OpenPose (OP), MediaPipe (MP), rehabilitation, tree structure skeleton image (TSSI), tree structure skeleton color image (TSSCI), variational auto encoder (VAE), Siamese twin neural network, simulator, human body movements, human pose estimation (HPE), computational imagination, computational creativity

## Abstract

In this article, we introduce a new approach to human movement by defining the movement as a static super object represented by a single two-dimensional image. The described method is applicable in remote healthcare applications, such as physiotherapeutic exercises. It allows researchers to label and describe the entire exercise as a standalone object, isolated from the reference video. This approach allows us to perform various tasks, including detecting similar movements in a video, measuring and comparing movements, generating new similar movements, and defining choreography by controlling specific parameters in the human body skeleton. As a result of the presented approach, we can eliminate the need to label images manually, disregard the problem of finding the start and the end of an exercise, overcome synchronization issues between movements, and perform any deep learning network-based operation that processes super objects in images in general. As part of this article, we will demonstrate two application use cases: one illustrates how to verify and score a fitness exercise. In contrast, the other illustrates how to generate similar movements in the human skeleton space by addressing the challenge of supplying sufficient training data for deep learning applications (DL). A variational auto encoder (VAE) simulator and an EfficientNet-B7 classifier architecture embedded within a Siamese twin neural network are presented in this paper in order to demonstrate the two use cases. These use cases demonstrate the versatility of our innovative concept in measuring, categorizing, inferring human behavior, and generating gestures for other researchers.

## 1. Introduction

This paper is an extended, updated version of the pHealth 2022 conference publication [1]. It presents a more general and generic approach to the solution based on a super-object model using a TSSCI image. We improved the model by basing it on variational auto-encoder (VAE) for generating human movements instead of the generative adversarial network (GAN) model presented at the pHealth 2022 conference. Additionally, we explain how to control the skeleton choreography movement in the simulation components.

We present a more effective CNN network compared to the CNN network presented at the conference. Furthermore, we expand our explanation of the dataset preparations, pre-processing, data structure, and the meaning of the various database types. We also refer to various practical challenges, such as normalizing the human skeleton, improving the loss function, providing more detailed reference to the movement performance scoring, and adding analysis and explanation of how the network operates using the t-distributed stochastic neighbor embedding (t-SNE) algorithm.

We update and expand our experiment descriptions and present better results than the ones previously presented at the conference. Overall, this article presents a significantly improved and more comprehensive version of the research presented at the conference, with additional details, improvements, and explanations.

## 2. Literature Review

Remote healthcare utilizes human posture and gait for real-time medical rehabilitation [2,3]. The COVID-19 pandemic demonstrated the importance of remote diagnosis and treatment. This importance of remote healthcare is further emphasized in the introductory paper of the Frontiers in Medicine Research [4]. The comprehensive view presented in this paper underscores the significance of lifestyle factors, including exercising, in healthcare management. By incorporating these principles into remote healthcare practices, healthcare professionals can enhance patient care and optimize treatment outcomes.

In the modern age, it is now possible to utilize a camera video stream to collect, analyze, and interpret human emotions in a remotely located 3D environment by using artificial neural networks [5]. Our objective is to characterize human motion using neural network architectures such as auto-encoder [6] and Siamese twin [7], in conjunction with human pose estimation (HPE) techniques such as real-time multi-person key point detection algorithms such as OpenPose [8] and MediaPipe [9]. Remote therapy may be used when many patients recuperate after movement disorders caused by hip, knee, elbow, or shoulder surgery [10,11]. A variety of non-contact medical treatments might be developed by utilizing a family of neural network designs resulting from this research. This dissertation proposes a solution to enrich and enhance skeletal data veracity, by providing accurate and specific data tailored to research requirements using the VAE deep-learning method [12]. In the articles [13,14,15], some databases contain video clips of human movements divided into a variety of classes. They start by processing the data using the OpenPose software, translating the video frames into skeletal pose sequences, which are then analyzed. A three-dimensional matrix represents each skeletal pose. To preserve the relationship between the skeletal joints, the authors reordered every pose as part of deep first search (DFS). Our movement generator is based on skeletal data that provide spatial and temporal information. Several studies have investigated the issue of recognizing human movement using skeleton-based neural networks (CNNs) [13,16]. Therefore, deep convolutional generative adversarial networks (DC-GANs) use CNN layers as their generator and discriminator [8]. It is proposed in [13,15,17,18] to use an image format (TSSI—tree structure skeleton image) to generate a tree structure skeleton image based on the collection of N tree structure sequences. Therefore, we utilized deep first search (DFS) to restructure and create tree structure skeletons.

## 3. Materials and Methods

**Figure 1:**
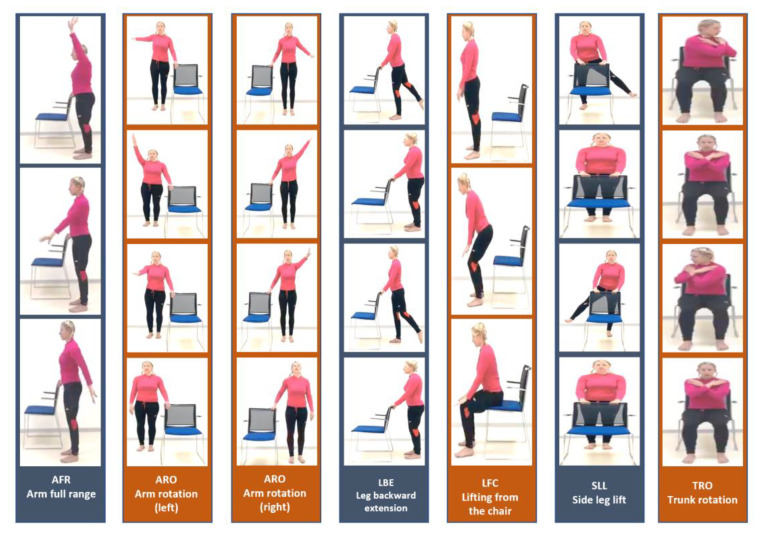
Six basic physiotherapy exercises that we developed and recorded.

There are six basic physiotherapy exercises in the database, which have been carefully selected to be suitable for analyzing and processing with a single camera (two-dimensional processing see [3]), as illustrated in Figure 1.

**Figure 2:**
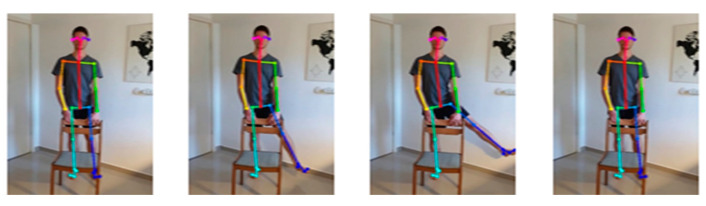
Database has been encoded as skeletons—a skeleton per frame.

There are approximately 100 participants in our proprietary database, which is now open to the public. Each participant performs six exercises. Ten cycles comprise each exercise (e.g., rotating the right arm). Exercises are performed once with a right tilt and once with a left tilt (for example, once with a right foot rotation and once with a left foot rotation). A total of about 7500 motion cycle videos have been tagged and timed in the database. This study included healthy subjects (volunteers—students) with no disability identified during tests to control postural stability. The subjects group comprised of 4 men and 26 women with an average age of 21.1 (standard deviation (SD) 1.2) years, body weight 64.8 (SD 9.4) kg and body height 170 (SD 9) cm. One single measurement of each subject was taken during the session. The study was performed in accordance with the Helsinki Declaration and the study protocol was approved by the local Ethical Committee, by the Faculty of Biomedical Engineering, Czech Technical University in Prague. The entire database has been encoded as skeletons—a skeleton in every frame (see Figure 2).

**Figure 3:**
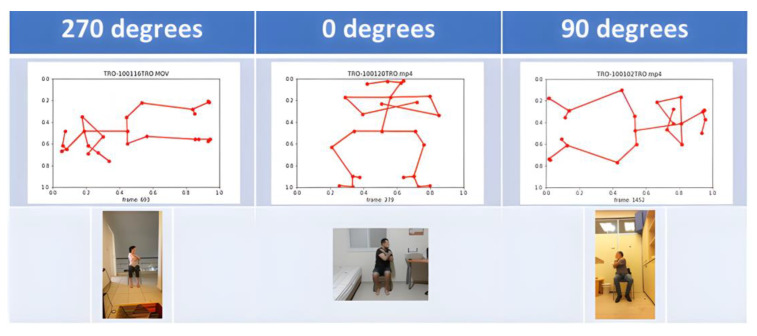
Homemade photoshoot challenges due to camera orientation.

Performing exercises creates skeletal structures. The human body is represented by 25 vertices in each skeleton. The vertex has three components: Coordinate X, coordinate Y, and coordinate C, which indicates the level of certainty about each point in the skeleton on a scale from 0 to 1 (1—absolute certainty, 0 absolute uncertainty). Collecting data for training a deep learning model requires careful consideration of various factors that could potentially affect the quality and reliability of the data. The fact that the students at Ben Gurion University were photographed independently in their homes simulates a real-world scenario but also introduces data variability and quality challenges. The difference in resolution and orientation of the photographs is a significant concern as it can impact the performance of the deep learning model. The four different orientations observed—head touching the upper edge of the monitor, a skeleton lying on the right side, upside down, and a skeleton lying on the left—could potentially affect the accuracy of the model predictions (see TRO exercise as example in Figure 3). To mitigate this issue, we had to perform data pre-processing by rotating the skeletons to a standard orientation (0 degrees).

### Skeleton Rotation

We extract n key points per skeleton from a single video frame. The frame may contain more than one skeleton, but we always use the first skeleton.

For the i’th key point Vi = ($x_{i}$, $y_{i}$), let $x_{i}$ be the Vi, X component.

The following formula can be used to normalize coordinates to the range −0.5 to +0.5

(1) $$x_{ni}=\frac{x_{i}}{\left(M_{axX}-M_{inX}\right)}-\frac{M_{axX}+M_{inX}}{2\left(M_{axX}-M_{inX}\right)}$$

where:

$M_{axX}$—max($x_{i}$) for i∈ℤ,0≤i≤n
$M_{inX}$—min($x_{i}$) for i∈ℤ,0≤i≤n
$x_{ni}$—The normalized value of the skeleton’s X component key points $x_{ni}\in \mathbb{R},0\leq i\leq n$

The second step is rotation of 90°, 180°, or 270°. Let Vi be the original 2D key point vector with $x_{i}$, $y_{i}$ components, which indicates the position of the key point prior to rotation. Based on the angle of rotation, we can use the following formulas to rotate a 2D vector: 90 degrees counterclockwise rotation: Ki = ($y_{i}$, −$x_{i}$); 180 degrees counterclockwise rotation: Ki = (−$x_{i}$, −$y_{i}$); 270 degrees counterclockwise rotation: Ki = (−$y_{i}$, $x_{i}$), where Ki is the rotated vector and $x_{i}$ and $y_{i}$ are the original components of the vector Vi. The third step is restoring the original coordinates. It is important to note that the frame is rectangular. Therefore, in a rotation of 270 degrees, X is the long side of the rectangle before the rotation, while Y is the long side after the rotation:

(2) $$x_{Ri}=\left(M_{axX}-M_{inX}\right)x_{n}+\frac{M_{axX}+M_{inX}}{2}$$

where $x_{Ri}$ is the restored coordinates after rotation.

The Y-axis of the skeleton should be similarly adjusted.

Another challenge introduced by data collection was camera vibrations and movement during training. As a result, we obtained a blurry or unstable image, affecting the performance of the deep learning model. To minimize the difficulties arising from unsupervised photography in the patient’s home environment, we recommended ensuring that the cameras used for data collection are stable and that the physiotherapist instructs the patients to minimize camera movement during data collection. In conclusion, collecting high-quality data is crucial for the success of deep learning models in physical therapy applications. Careful consideration of resolution, orientation, and camera stability are essential to ensure that the model’s predictions are accurate and reliable.

## 4. Human Movement as a Static Super Object

### 4.1. Review of Existing Technics to Describe Human Movements

We can use recorded series of human body positions using motion capture to represent a series of human body positions. Motion capture refers to recording a person’s movement while wearing a marker or sensor and then using those data to produce an animation of that person’s movement (the animation shows the movement of specific dots on the human body). The animation result may either be a single image or a series of images. Pose estimation is another method for representing the successions of human body postures at a specific time, which involves identifying and tracking the body parts and joints of a person in a video or sequence of pictures and encoding that information as a single image or a group of images. Motion representation is the representation of the motion of an object or a succession of moving objects in a meaningful and compact manner. Here are some examples:

- Optical flow describes the mobility of pixels or points in an image series by estimating their displacement between consecutive frames [19];
- Researchers use Euler angles and quaternions to represent an object’s orientation in 3D space by establishing rotation angles around the x, y, and z axes [20];
- By charting an object’s position in space at different points in time, researchers can use a trajectory to describe an object’s passage through time [21];
- In motion fields, researchers store the velocities and accelerations of each point on the object to represent an object’s motion over time [22].

### 4.2. Our New Approach-Movement as a Static Super Object

Research literature analyzes the movement as an object in motion, meaning a sequence of objects’ positions, orientations, and sizes that change over time and space. Our innovative approach combines objects and represents the entire movement as a single static super object. Our original approach provides a fresh perspective on human movement. To begin with, we treat a tree structure skeleton image (TSSI) as a color image (TSSCI), then generalize movement to a color image as an object. For example, if there are several objects in the picture (such as three cats in a typical picture), then in our model there are three movements, and therefore there are three objects within the TSSCI image. By applying this approach, an object can be small, meaning the object begins and ends simultaneously within the image. In our representation a small object indicates fast motion as opposed to a large object, which describes slower motion. As a result of adjusting two object sizes, we can sync them up. We can locate the object in a specific place in the TSSCI image while identifying all other pixels as the background. The places where the object does not exist in the image indicate no movement or an idle movement period before and after the exercise of interest. Using this concept, we can automatically determine when a movement starts and ends, and therefore we can use automatic editing to extract specific movements from long videos. Neural networks can extract unique attributes of objects in the latent space, thus allowing the network to differentiate between objects in the same way that describing the movement as a super object allows extracting movement characteristics.

### 4.3. Generic Neural Network Implementations with TSSCI

We can use TSSCI images as inputs to all neural networks capable of analyzing and processing images. For example, a CNN classification network can label objects within a color image, such as tagging cats. Inserting a TSSCI image into the CNN, the network can identify and tag different movements. We can generate TSSCI images using variational auto-encoder (VAE) networks containing new super objects, i.e., simulating new fake movements. Using the VAE network, TSSCI allows the generation of objects, such as faces. In addition, the VAE network allows the combination of objects (faces). In order to produce objects A and B together, one can take a picture of a bald man without glasses (object A) and combine it with another picture of a man wearing glasses and hair (object B). Thus, it is possible to produce the man (object A) with hair and glasses that we take from object B. In the same way, we can take a TSSCI image of a periodic movement of raising hands up and down (object A) as well as a TSSCI image of a periodic movement of raising a leg (object B) and then generate a fake combination that describes a skeleton raising its hands as well as its legs.

### 4.4. How to Define and Label Specific Movement as a Super Object

Our article has explained the concept of representing motion as a static object within a TSSCI image. Next, we will examine how super objects are defined and labeled. It is not easy to classify movements. For example, how do we describe the physical exercise of raising and lowering hands? Is there one movement for raising and one for lowering a hand? Could the super object be a combination of two movements? In general, what does a super object look like? Can we even distinguish it or describe it in the TSSCI image? The first advantage of our approach is that we can define super objects for each partial movement and even connect them. However, movement classification and labeling is a subjective process as opposed to ordinary objects such as cats, which are generally agreed upon and, therefore, can be labeled. Moreover, the abstract nature of the TSSCI image makes it difficult to identify, locate, or define an object within the image as a human. For the same movement, we can obtain many different labels. In order to overcome this problem, we utilize the motion time domain, that is, by tagging the movement within a movie before converting it into TSSCI format. In other words, each individual will label the videos based on their understanding and objectives. We convert these tagged videos into TSSCI images. Thus, we have a labeled TSSCI image containing a super object that describes movement. Despite the difficulty of understanding the super object in TSSCI images, it is not necessary since the neural network can identify it. In summary, the user or the researcher determined the nature of super objects. Given a video that the user tagged, we converted it into a TSSCI image, and then we succeeded to tag specific super objects in the TSSCI domain. After collecting TSSCI images representing a motion or a collection of motions, it is possible to train the network to perform tasks such as motion tagging, generating new motions, or other options that the neural networks offer us.

### 4.5. TSSCI Use Cases Examples Base on Super Objects

For clarity and to demonstrate how extensively relevant our approach is to studying human body movements, we provide multiple interpretations for referring to the human movement as a super object using TSSCI. Table 1 illustrates how classical and advanced neural networks, commonly used for various image processing applications, can be applied to TSSCI images representing movement as a static super object.

By taking a novel approach to human motion, we show in Table 1 that deep learning networks can process the TSSCI and have a wide range of practical applications.

### 4.6. Comments Regarding Table 1

- This table presents a proposal for using existing architectures, but some modifications may be necessary, such as changing the dimensions of the input image. A TSSCI image typically has smaller dimensions than a typical image input. As an example, in the examples we present later in this article, the dimensions of the TSSCI image are 49 × 49 × 3, thus using the VGG network for classification was not appropriate since the image dimensions at the VGG entrance are 240 × 240 × 3, and the depth of the network exceeds our database size. Therefore, overfitting occurred. For this reason, we used an EfficientNet-B7 image classification CNN network. Similarly, particular adaptations will be required to use the architectures listed in the table. We recommend selecting most suitable networks for performing the desirable task and scaling the TSSCI dimensions;
- A few propositions require practical proof because they are theoretically logical inferences. We must train the network extensively with diverse TSSCI images tagged with various texts to formulate a choreography in which DALL-E uses a ritual to control human skeleton movements;
- We recommend using this table to develop additional ideas based on existing architectures, using the super object method for human movement description.

## 5. Convert a Sequence of Human Skeleton Movements into a TSSI Single Image

**Figure 4:**
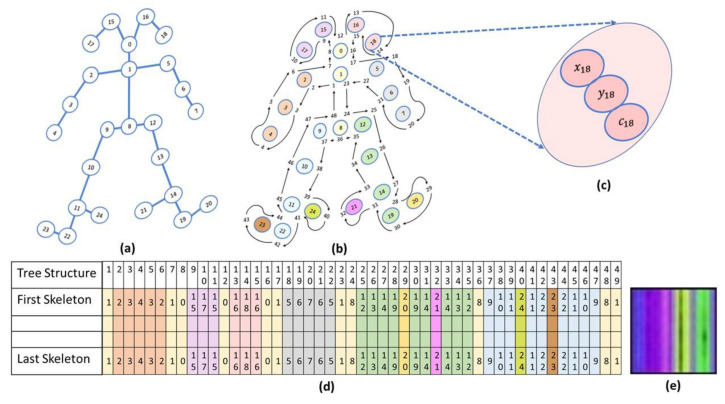
OpenPose Tree structure skeleton to TSSCI: (**a**) OpenPose skeleton; (**b**) Skeleton tree for TSSI or TSSCI generating; (**c**) Visualizing key points: A 3D representation; (**d**) A collection of tree structures: pose patterns are tabulated row by row; (**e**) Visualizing key point locations and confidence Levels: TSSCI-RGB Image.

Paper [14] presents a method for recognizing human actions in video sequences using a combination of spatial-temporal visual attention and skeleton image processing. In the paper, the authors introduce the concept of converting the skeleton image sequences into tree structure skeleton images, which they refer to as “TSSI” images. TSSI images are a type of abstract image representation that captures a person’s skeletal structure in a video sequence and can be used to analyze and recognize human actions (see Figure 4).

The authors show that TSSI images are a more efficient and effective representation of human actions compared to traditional video or image data, as they capture the critical aspects of movement and can be processed more efficiently. To recognize actions in TSSI image sequences, the authors propose a method that combines spatiotemporal visual attention with a convolutional neural network (CNN) for classification. They used the visual attention mechanism to focus on relevant parts of the TSSI image sequence and the CNN to recognize the exercise performed by the athletes. In contrast to the traditional TSSI perspective, in which TSSI represents a movement within an image, our TSSCI (tree structure skeleton color image) method first converts the key points into RGB color images (we normalize the skeleton coordinate values to be between 0 to 1). Converting skeleton key points to RGB-colored TSSCI images allows for representing multiple human movements as one super object. X, Y, and confidence level—C coordinates represent each key point in the skeleton. We grouped the skeletons in three dimensions array. We convert the 3D array into RGB channels by taking the red color channel to represent the X coordinates, the green color channel to represent the Y coordinates, and the blue color channel to represent the C coordinates. We refer to the colored TSSI as TSSCI, which represents the composition of the exercise from start to finish as an abstracted color image.

### 5.1. TSSCI Needs a Buffer to Convert Temporal-Spatial Data into Spatial Data

With no prejudice to generality, TSSCI contains a sequence of our human skeletons, initially presented as time series (e.g., each video frame might contain one or more skeletons). To convert video into a TSSCI image, we need a frame buffer containing frames with or without skeletons. The buffer contains a time mark for the first and last frames. As a result, when processing skeletons, we have complete information about the entire movement from beginning to end. Several factors determine the buffer size: the degree of latency we are willing to accept between real-time (the last frame in the buffer) and how much historical information we require (the first frame). Image resolution is also a factor. Our column resolution component is determined by how many key points are in our skeleton, which we enlarge by the TSSCI skeleton tree. (For example, the OpenPose body model contains 25 key points. By duplicating some key points following the skeleton tree scheme, we obtain 49 key points, which constitute 49 columns.). The row resolution component is determined by how fast the frame rate is and how long the movement (or submovement) is. We can break apart any length movement (or submovement) into slices using our super object approach. Furthermore, with our super object concept, we can process each sub-super object separately and then concatenate them to form one super object. This approach can also accelerate the processing speed leveraging GPU parallel processing.

### 5.2. Methods for Normalizing Skeleton Coordinates for the Implementation as TSSCI Pixels

As explained in the previous section, we have a buffer that contains all the movement of the skeletons from the beginning to the end. In order to prevent discontinuities of the skeletons between frames as a result of normalization, we will simultaneously normalize the entire buffered group of skeletons. Several normalization methods are described in the literature. For TSSCI, we can simultaneously measure the reference points of all the buffered skeletons using any of the following methods.

- Mean and standard deviation: Normalizing the coordinates by calculating their mean and standard deviation [23];
- Min-Max normalization: We can use it to scale coordinates so that they fall within a specified range, such as (0, 1). The minimum and maximum values of the coordinates must be determined first, and then the coordinates must be scaled using the following formula: (x − min)/(max − min) [24];
- Zero-mean normalization: Center the data around zero by subtracting the mean from each coordinate. This method helps remove any bias in the data [25];
- Root mean square normalization (RMSE): To ensure that subsequent analyses are not affected by the scale of the coordinates, this method scales the coordinates so that the root mean square (RMS) is equal to one [26];
- Scaling to unit norm: Scaling coordinates in this manner ensures that the L2 norm of the coordinates is equal to one. It ensures that scale does not affect the results of subsequent analyses [27].

We present several examples of using the super object method in this article. For the results presented in this article, we used the Min-Max normalization method. We followed the following formulas:

If i is the row index in the TSSCI and j is the column index in the TSSCI and the $C_{ij}\geq threshold$ (if the confidence level is low then the *x*, *y* coordinates are considered as noise):

(3) $$\begin{array}{l} x_{max}=max\left(max\left(x_{ij}\right)\right)\\ x_{min}=min\left(min\left(x_{ij}\right)\right)\\ x_{L}=x_{max}-x_{min} \end{array}$$

(4) $${\hat{x}}_{ij}=\frac{x_{ij}}{x_{\begin{array}{c} L \end{array}}}$$

In the same way we calculate the normalized y component:

(5) $${\hat{y}}_{ij}=\frac{y_{ij}}{y_{\begin{array}{c} L \end{array}}}$$

The normalized key point ${\hat{kp}}_{ij}$ is defined as:

(6) $${\hat{kp}}_{ij}=\left({\hat{x}}_{ij},{\hat{y}}_{ij},c_{ij}\right)$$

which used as our TSSCI pixel.

### 5.3. TSSCI Dataset Augmentation

The limited amount of video data in our database posed a challenge for training neural networks effectively. To address this, we applied dataset augmentation techniques to the videos. The videos were sampled at 30 frames per second, with a 33-millisecond interval between consecutive frames. This short time frame made it difficult to capture significant differences in movement from one frame to the next. Each video in the database had a typical length of 100 s. To ensure that our method for measuring and labeling movements based on TSSCI was effective, we selected a sample of 49 frames (approximately two seconds of separate consecutive frames), to obtain meaningful information between frames. We employed two modeling methods. The first method involved randomly selecting 49 frames out of the total number of frames, allowing for acceleration and deceleration within the exercise. The second method involved dividing the total number of frames into 49 equal segments and randomly selecting one frame from each segment. This method smoothed out fluctuations and internal accelerations within the exercise.

### 5.4. CNN-Based Automatic and Manual Video Editing

In order to improve the accuracy of the initial training of movements, it was necessary to eliminate unnecessary frames at the beginning and end of each video. To achieve this, we manually edited the videos and marked the frame numbers for the start and end of each movement in a separate CSV file. We also demonstrated that the TSSCI method is robust against the specific start and end location of the movement, as convolutional neural networks (CNNs) have the ability to identify the movement regardless of its location or size in the video, in much the same way as a CNN can identify a cat in an image despite its location or size within the image. To accommodate both approaches, we developed a code that allows the user to either work with the entire video without editing or with edited videos by specifying the start and end points using the CSV file, resulting in more accurate results.

### 5.5. Treatment of Low Confidence Level Key Points (Missing Key Points)

In some cases, the skeleton key point extraction software may fail to locate a key point with sufficient accuracy. This can be due to various reasons such as a person being photographed in profile, where one shoulder is visible while the other is obscured, or poor lighting conditions that deteriorate image processing quality. To address these missing key points, we implemented in our algorithm a method termed “complementing from the left”. This method replaces missing key points with their closest neighbors on the left. This results in an unrealistic representation of the skeleton where body parts appear to be suspended in the air without any connections. For example, if the elbow key point is missing, the algorithm will only show the skeleton up to the shoulder, resulting in a floating palm detached from the shoulder. Suppose there is a sequence of key points according to TSSCI description, for example 0, 1, 5, 6, 7, 6, 5, 1, 8, 12..., and key point 6 is missing (its confidence level is below a given threshold of 0.3). According to the left completion method, the sequence would be 0, 1, 5, 5, 7, 7, 5, 1, 8, 12... where 5 replaced the missing 6 and then 7 replaced the missing 6.

## 6. Movements Classification with Google CNN EfficientNet

**Figure 5:**
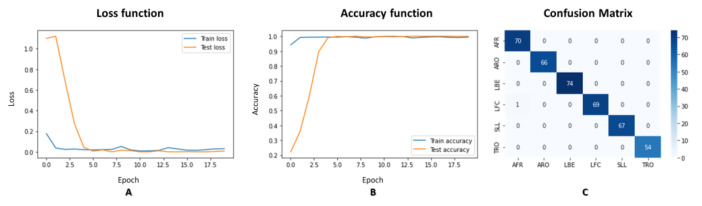
Classification with Google CNN EfficientNet results: (**A**) Loss function; (**B**) Accuracy function; (**C**) Confusion matrix.

We have explored our novel approach to analyzing human movements—the super object method. This method aims to provide a generic solution for a wide range of human movement analysis problems. To validate our theory, we experimented with demonstrating the effectiveness of the super object method. We used a convolutional neural network (CNN) optimized for small images with a resolution of 49 × 49. We used for our CNN classification model the EfficientNet-B7 classifier architecture, which has achieved state-of-the-art results in image classification tasks. EfficientNet is a new method for scaling CNNs that considers both the depth and width of the network and the resolution of the input image. This method balances the trade-offs between accuracy, computational cost, and the amount of data required, making it a promising approach for improving the performance of CNNs in various tasks. We employed several techniques to improve our model learning and prediction performance, including data augmentation and transfer learning. In the transfer learning approach, we trained only the last layers of the EfficientNet network while freezing the first three layers. It allowed us to achieve improved performance while minimizing the risk of overfitting to the limited data available. We set the final model to have six classes corresponding to the six different human movements that we have in our dataset: “AFR,” “ARO,” “LBE,” “LFC,” “SLL,” and “TRO.” We present, in Figure 5, the results of the classification and the training progress. Part A and Part B show the progression of the loss function and accuracy function, respectively, as a function of the number of epochs. Using a pre-trained network and a clean data set significantly contributed to fast and accurate learning. The rapid decline of the loss function and the steady increase in the accuracy function demonstrate the effectiveness of the super object method in human movement analysis. We summarized the classification results in the confusion matrix illustrated in Figure 5 The results of our experiment provide evidence that the super object method is a viable and practical approach to analyzing human movements.

As a final note, it is essential to highlight that for simplicity, we used only the dataset of the Czech students in this experiment. These students made exercise recordings in a controlled manner and under laboratory conditions, providing a clean and consistent data set for demonstration purposes. The goal was to demonstrate the principle of operation using the super object method and not to focus on extreme cases that may require more training and possibly use more complex architectures. Using the Czech students data set allowed for a clear and straightforward demonstration of the super object method and its potential for human movement analysis.

## 7. Variational Auto Encoder (VAE)

**Figure 6:**
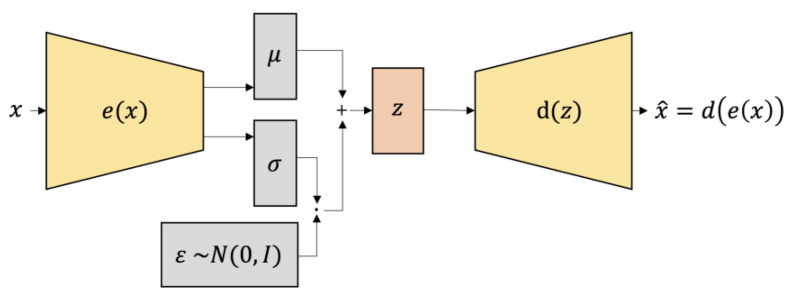
Structure of the variational auto-encoder (VAE).

A variational auto-encoder (VAE) is a non-supervised artificial neural network (see Figure 6). We design VAEs to learn a compressed representation of high-dimensional data, such as images or audio, and then utilize it to generate new data samples. As a generative model, VAEs can generate new instances of the input data distribution that they were trained on. In paper [12] Kingma and Welling describe the VAE framework as a Bayesian way to learn latent variable models. They offer a way to train the VAE by combining the encoder-decoder network with a variational inference objective.

The encoder network takes a sample as input and creates parameters that determine a probability distribution over the latent space. The decoder network takes a sample from this distribution and builds an output similar to the original input. The goal of variational inference is to find the parameters of the encoder and decoder networks so that the distribution in the latent space matches a distribution from before and the reconstructed output matches the original input. Another benefit of VAEs is that we can use the learned distribution over the latent space to generate new samples. By taking samples from the distribution it has learned, the VAE may be able to make new outputs that are identical to the original data. The ability of VAEs to generate new samples from the learned distribution makes them an efficient tool for creating images and sounds. Another feature of VAEs is their ability to learn deconstructed representations of incoming data. The fact that each dimension of the latent space corresponds to an essential feature of the input data, such as the position of an object in a photograph, demonstrates the usefulness of VAEs. This property makes VAEs useful for data compression and visualization applications. VAEs can learn compressed representations of high-dimensional data, generate new samples of the input distribution, and learn unconfused representations of the input distribution. Because of its capacity to combine encoder and decoder networks with a variational inference objective, the architecture presented in Kingma and Welling’s study [12] has become a popular method for training VAEs.

## 8. Demonstration of How to Measure Gesture Mimics Via Siamese Twin Neural Network

The neural network chosen for this project is the Siamese twin neural network [7]. The reason for selecting the Siamese twin network is its one-shot learning capability. The result is that once the network has been properly trained, it is possible to classify a new image into a class that was not included in the initial training. Using TSSCI technique, we managed to capture the entire motion of the human body in one image as a super object. It is not necessary to use all of the frames within a time window to create a good representation of TSSCI. We conclude that different gestures require different time windows for optimal TSSCI representation.

**Figure 7:**
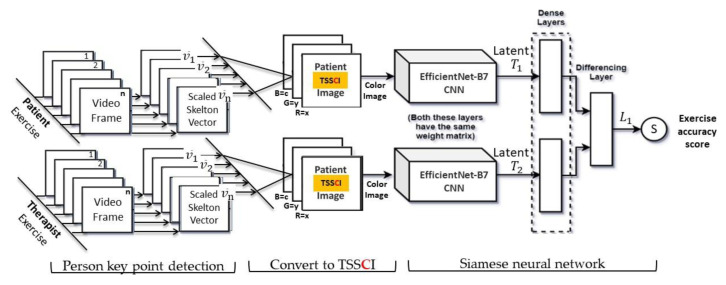
Siamese twin network layout.

The input to the network (see Figure 7) consists of a pair of TSSCIs with dimensions of 49 × 49 pixels each. Inputs are fed into the same convolutional and pooling layers and the output is a tensor with 4096 elements for each input, which can be considered as a type of code or latent of the TSSCI. For the CNN block in the Siamese twin model we have used the EfficientNet-B7 [28] classifier architecture embedded within a Siamese twin neural network. “EfficientNet-B7 achieves state-of-the-art 84.4% top-1/97.1% top-5 accuracy on ImageNet, while being 8.4× smaller and 6.1× faster on inference than the best existing ConvNet.” [28]. These latent codes are fed into the differentiation layer, which computes their $L_{1}$ distance.

### EfficientNet Transfer Learning

To improve the learning and prediction performance of our model with limited data, we employed several methods. One method was data augmentation. Another method was taking advantage of the pre-trained EfficientNet network for image classification by performing transfer learning. In our approach, we kept the parameters of the first three layers of the network, as we considered that these layers are responsible for learning the background information of the objects, which is common across different object types. This allowed us to focus the training on the last layers, which were tasked with learning the specific features of our motion object. By doing this, we aimed to achieve improved performance while minimizing the risk of overfitting to the limited data available. We can train the Siamese twin neural network using two different methods. The first is a complete training of the entire network from start to finish, which is suitable for adding a new movement to the database that does not require any relation or connection to other movements. The second method is a particular case of the first, which we term the partial Siamese twin network. This method calculates an accuracy score when comparing a reference movement (performed by a trainer/therapist) to the patient movement. The Siamese twin network is a classic approach to measuring similarity between a pair of images, and this is an unsupervised problem, as the training is based solely on labeling similar and dissimilar movements. The second method is a classic supervised problem, where the movements are known in advance and labeled according to the recognized movement the patient is performing. In this case, we only train the CNN as a closed classification system to classify the known movements.

(7) $$L_{1}=D=flatten(\left|T_{1}-T_{2}\;\right|)$$

where $T_{1},\;T_{2}$ are the tensors obtained from the convolutional and pooling layers (latent feature vectors), respectively. There is only one neuron in the final dense layer that has a sigmoid activation function. We can model this layer mathematically:

(8) $$L_{1}=\sigma (bias+\underset{i}{\sum }\left(w_{i}C_{i}\right)\;Loss=\left(1-Y\right)\frac{1}{2}{\left(D_{w}\right)}^{2}+\left(Y\right)\frac{1}{2}{\left\{\max\left(0,m-D_{w}\right)\right\}}^{2}$$

where:

- σ is the sigmoid function:

(9) $$\sigma \left(x\right)=\frac{1}{1+e^{-x}}$$

- $C_{i}$ is the i’th element of the input vector $C=Concat\left(T_{1},\;T_{2}\right)=\left[T_{1},\;T_{2}\right]$;
- $w_{i}$ is the corresponding i’th weight.

(10) $$Loss=\left(1-Y\right)\frac{1}{2}{\left(D_{w}\right)}^{2}+\left(Y\right)\frac{1}{2}{\left\{\max\left(0,m-D_{w}\right)\right\}}^{2}$$

where:

- *Loss*: overall loss that the model incurs in making predictions for a binary classification problem;
- *Y*: label for a particular data point. It takes a value of 0 or 1 depending on whether the data point belongs to class 0 or class 1;
- $D_{w}$: difference between the predicted value and the actual label. It represents how well the model is performing on a particular data point;
- *(1 − Y)*: error incurred by the model when it predicts the negative class;
- ${\left(D_{w}\right)}^{2}$: square of the difference between the predicted value and the actual label. It is used to penalize larger differences;
- *(Y)*: error incurred by the model when it predicts the positive class;
- max(0, *m* − $D_{w}$): margin between the predicted value and the actual label. The max function ensures that this value is always non-negative;
- *(1/2)*: term used to normalize the loss.

Accordingly, the output of the network is a number between 0 and 1, which correlates to the degree of similarity between the two inputted TSSCIs. The closer the output value is to zero, the higher the level of similarity predicted. Our Siamese twin network output termed $L_{1}$, ranges from 0 to plus infinity. Therefore we converted the $L_{1}$ into an accuracy score S, which ranges from 0 to 1, with 0 being a complete mismatch and 1 being an exact match. We used the following formula to normalize the $L_{1}$ score to the S score:

(11) $$S=1-L_{1}/\|T_{2}\|\;$$

where:

- $\left\|T_{2}\right\|$ is the therapist’s latent norm, our reference movement that the CNN converts from the TSSCI exercise image into a latent vector.

## 9. Results

### 9.1. Creating the Extended Database Using Normalization and Augmentation

**Figure 8:**
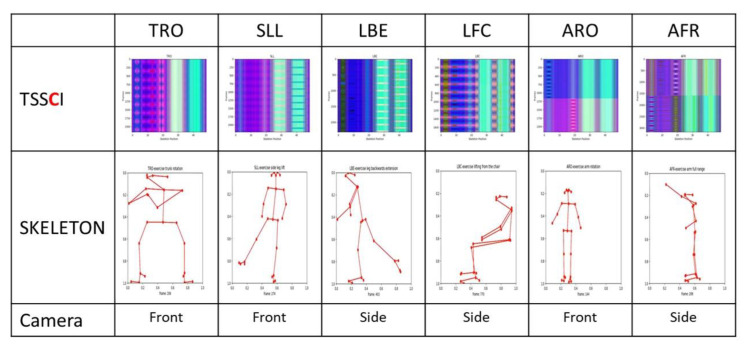
Extended database using normalization and augmentation.

We utilized the EfficientNet-B7 network to classify six pre-defined movements from a database containing 100 students, each performing six movements. We used OpenPose to extract the skeleton vectors from the video frames into NumPy arrays (the skeleton key points vector extracted from a video frame is a line in the array) and performed a centering operation to place the skeleton in the center of the frame. The x and y coordinates were normalized to values between 0 and 1, while the level of confidence c remained between 0 and 1. Due to the low confidence level values for some key points, we could not rely on the position evaluation values provided by OpenPose. Instead, we used the “complementing from the left” algorithm. For instance, if the elbow key point were missing, the algorithm would only display the skeleton up to the shoulder, resulting in a floating palm in the air separate from the body. We selected 49 random lines from each normalized NumPy file for augmentation. Those 49 lines are equivalent to 49 frames with an interval between frames of approximately two seconds. We used them to create a TSSCI tensor, an RGB color image where the red channel represents x values, the blue channel represents y values, and the green channel represents the confidence level c. We repeated this operation 2004 times to generate a total of 2004 tagged TSSCI images. We used 1603 images for training (80%) and the remaining 401 (20%) for evaluation. Figure 8 contains some examples from our extended dataset using normalization and augmentation. The following table shows TSSCI and one of its single skeletons. We took the examples provided here from the videos of the physiotherapist. The expert is our source of reference when performing a correct exercise.

Figure 8 shows TSSCI images for six exercises: TRO—trunk rotation; SLL—side leg lift; LBE—backward leg extension; LFC—lifting from the chair; ARO—arm rotation; AFR—arm full range; In TSSCI images that describe exercises ARO and AFR, there is a central contrast line. There is a separation of tones between the upper and lower portions of the image. This is due to the fact that the movements in these two exercises are performed in two parts: the first involves exercising the right side of the body cyclically, while the second involves exercising the left side of the body cyclically. There are 10 consecutive movements cycles in each part.

### 9.2. Train and Evaluate the EfficientNet-B7 Model on TSSCI Images

**Figure 9:**
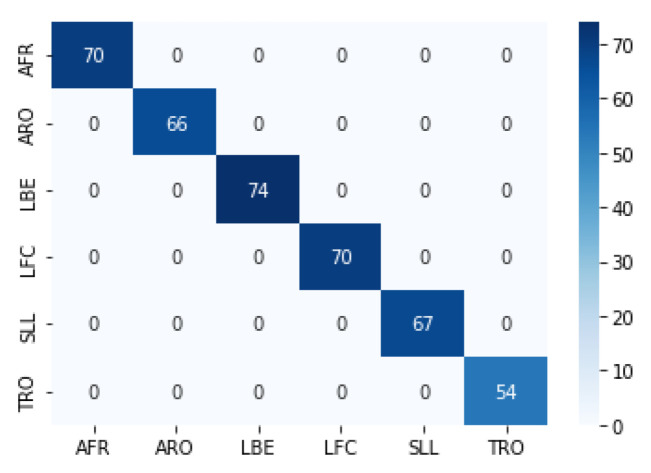
Evaluate the EfficientNet-B7 model on TSSCI images via confusion matrix.

We utilized the pre-trained EfficientNet-B7 network for image classification by performing Transfer Learning. We set the network to have six outputs corresponding to the six movements, with an evaluation set that contains: 70 images for AFR, 66 images for ARO, 74 images for LBE, 70 images for LFC, 67 images for SLL, and 54 images for TRO. The EfficientNet-B7 network yielded a probability vector with six components for each movement, summarized into one (100%). We converted a movement video into a TSSCI image to perform the movement detection, which was then input into the trained EfficientNet-B7 network. We determined the predicted movement label by selecting the output with the highest probability. The classification performance was measured using an evaluation set of 401 samples. The results were presented in a confusion matrix, as shown in Figure 9.

**Figure 10:**
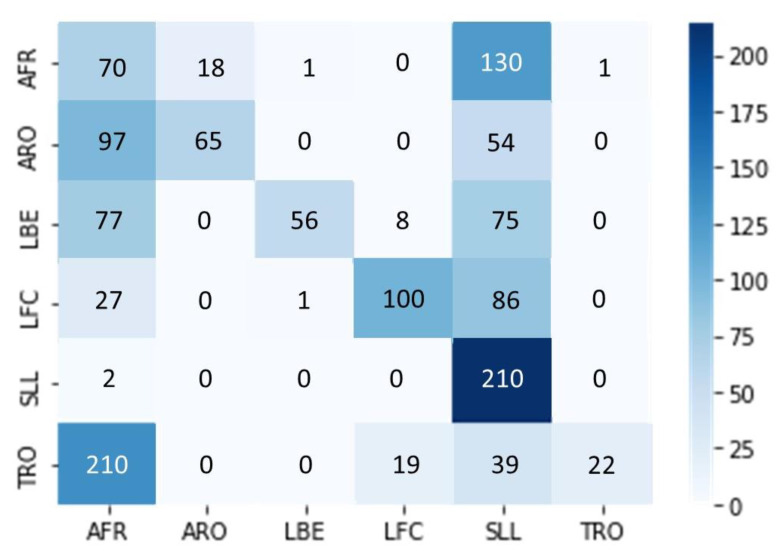
Classifying exercises with the super object under challenging conditions.

We conducted classification of a noisy data set consisting of exercises performed by students from Ben Gurion University in a home environment. The BGU students recorded these exercises under challenging conditions with varying cameras, shooting distances, camera movement, and lighting. We did not edit the BGU student videos; therefore, each exercise starts and ends at different times. We evaluated the results by presenting them in a confusion matrix, which summarizes the network predictions for each exercise (see Figure 10). The network output is a probability vector for each of the six exercises, and the chosen exercise has the highest probability. The diagonal of the matrix shows how many times the network correctly predicted the exercise. Despite the difficult conditions and although each exercise begins and ends in a different frame, the results show that the network was able to classify the exercises successfully since the diagonal of the matrix is dominant. When using the super object method, we treat the movement as an object, allowing the network to classify movements effectively even in diverse shooting conditions.

While it is possible to continue training and improving the network to achieve better results, that is beyond the scope of this article. The goal is to demonstrate the effectiveness of the super object method in referring to movement.

### 9.3. Results with Variational Auto Encoder

**Figure 11:**
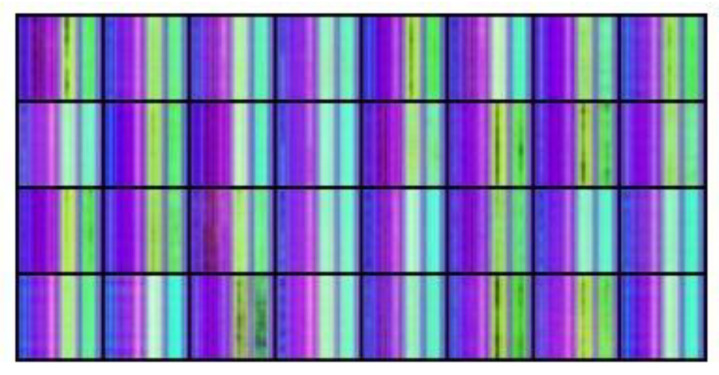
32 Fake TSSCI images that were generated with AVE.

As a demonstration that our method is general and does not depend on the algorithm of a particular generator, we are presenting results from another movement generator, this time using variational auto-encoder (VAE). The results prove that our method is general and does not depend on the algorithm of a particular generator. This architecture was presented by Kingma and Welling in their paper [12]. The authors describe VAE as a generative model trained using variational principles, which presents an unsupervised learning approach. There are two main components to the VAE architecture: an encoder and a decoder. It is possible to illustrate the architecture using diagrams, as shown in Figure 6: The encoder converts input data into a latent representation, and the decoder converts that latent representation back into the original data space. With our TSSCI images dataset, we trained the VAE and generated several fake TSSCI images (see Figure 11).

**Figure 12:**
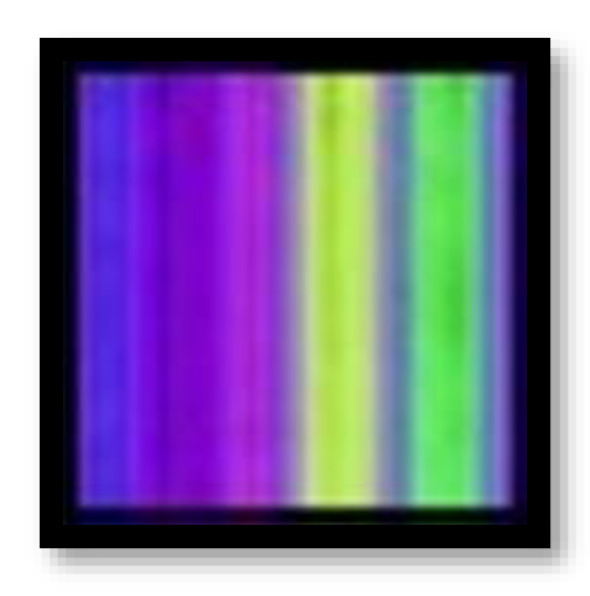
The display of TSSCI A image out of 32 generated by VAE.

**Figure 13:**
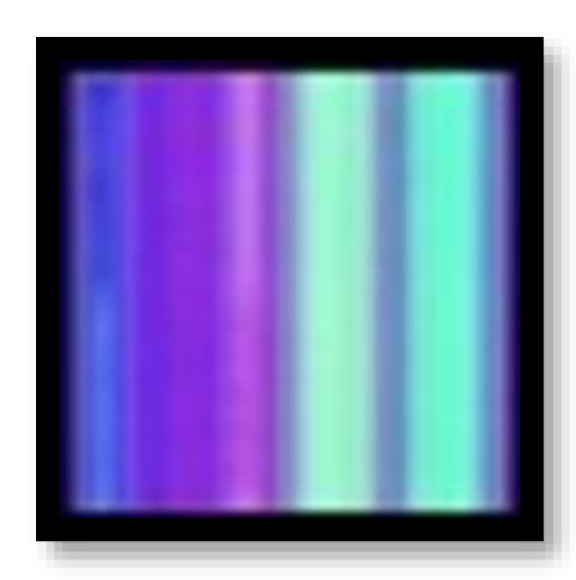
The display of TSSCI B image out of 32 generated by VAE.

**Figure 14:**
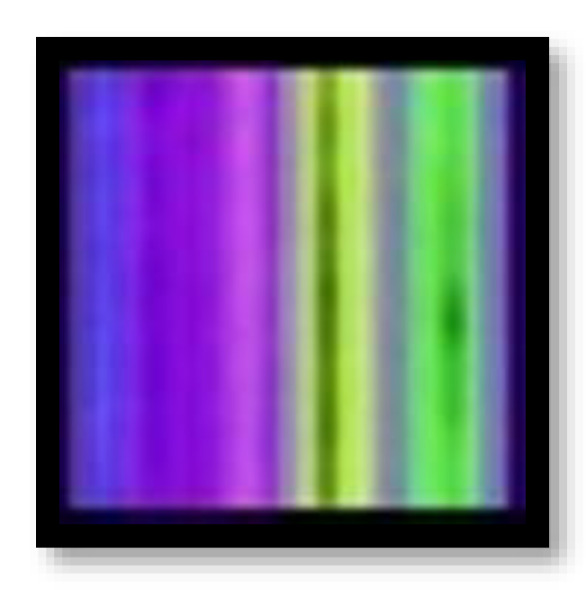
The display of TSSCI C image out of 32 generated by VAE.

**Figure 15:**
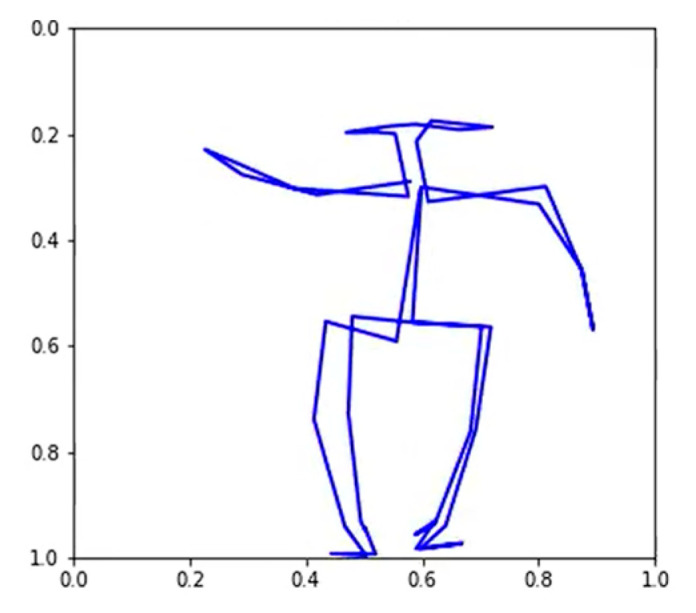
A small skeleton performs movement A (two hands).

**Figure 16:**
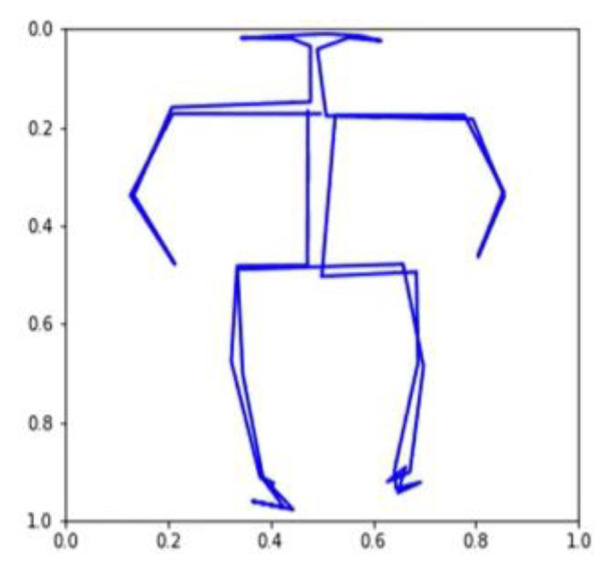
A tall skeleton performs movement B (right hand movement).

**Figure 17:**
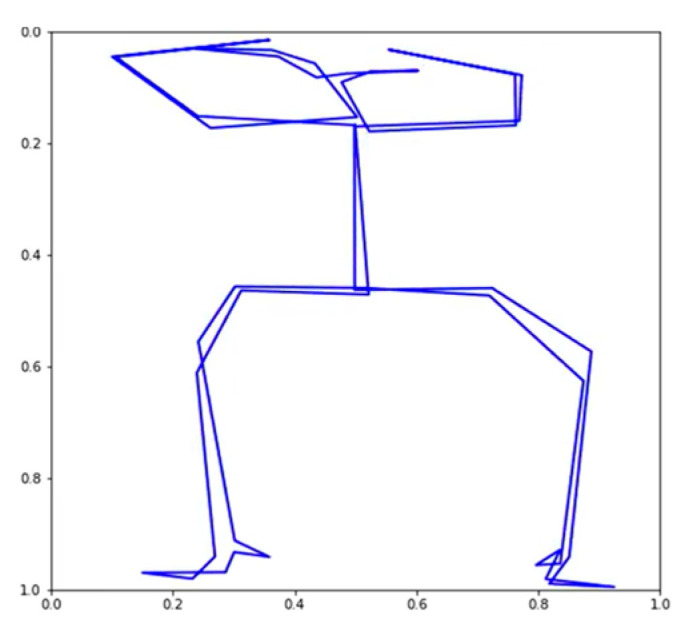
A tall skeleton performs movement C (hands up).

Using the VAE, we were able to produce 32 TSSCI images (Figure 11). Out of 32 images, we randomly selected three (see Figure 12, Figure 13 and Figure 14). We converted the three images back into the time domain to reproduce the skeleton movement generated by the network. In Figure 15, Figure 16 and Figure 17, we present a frame sample containing one skeleton from each TSSCI. We converted each skeleton vector (each line in the TSSCI image) into a video frame sequence that contained one skeleton. In each one of the three pictures we preset one frame from the movement sequence. In Figure 15, we see a small skeleton performing two hand movements over a sequence of frames. Each TSSCI generated by the network is essentially another exercise that describes skeletal movement over a sequence of frames. In Figure 16 we can observe a tall skeleton performing a right-hand movement, and in Figure 17 another tall skeleton performing a hands-up movement. According to the method of representing the skeletons based on the tree structure skeleton image, some key points in the skeleton appear more than once in the vector representing the skeleton. As a result of applying the TSSCI method, some key points appear twice when compared to the original 25 OpenPose key points skeleton. This results in a skeleton vector consisting of 49 key points instead of the original 25 OpenPose key points skeleton. This structure is designed to ensure the connections between the points and preserve a structure of the logical human movement. In our case, we generate new TSSCI images; therefore, we generate new key points, but although initially some of the points are duplicated and identical, in our fake images, we obtain differences between the location of the identical points. As can be seen in the photos Figure 15, Figure 16 and Figure 17, the differences are minor. We can combine key points into one point, as we showed in VAE products (see [1]), but we chose to emphasize this point for the explanation.

**Figure 18:**
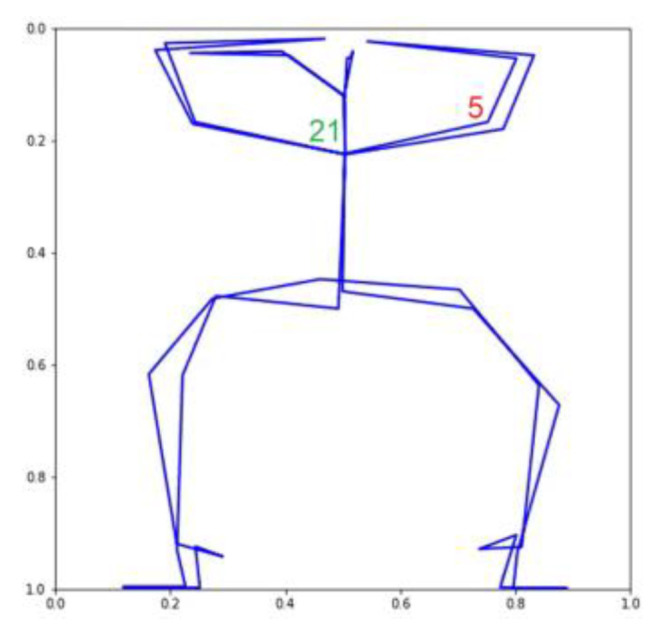
Merge only key point 21 while key point 5 remains unmerged.

**Figure 19:**
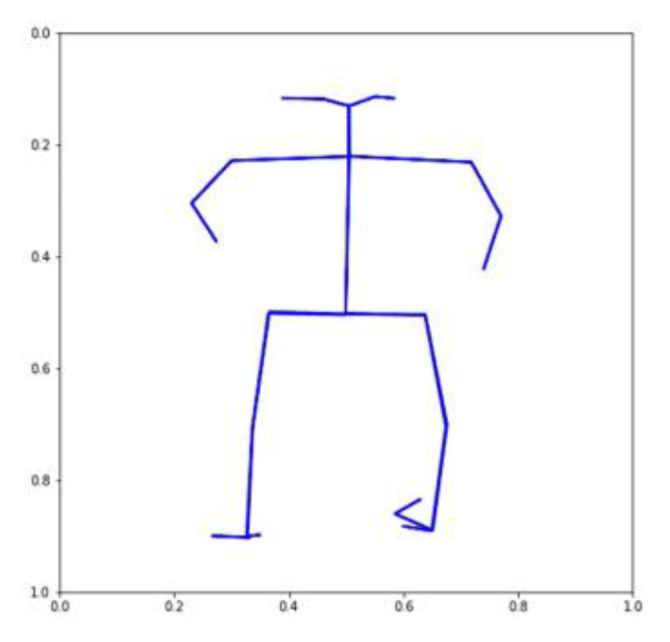
By adding the KPMSE to the loss function, key points are combined.

We show, for instance, how we can fix the same key points if we treat the motion in the image as an object (see Figure 18 and Figure 19).

Since we consider human motion an object in the image, we can use the loss function to constrain the network during training. In the VAE network, the loss function is composed of two components: the mean squared error (MSE) and the distance between the distribution of the training group and a normal distribution with mean 0 and variation 1, which we call the DKL.

(12) Loss=MSE+βDKL

Noting that a key point is a vector of three components (a 3D vector). It contains the coordinates X, Y, and confidence level C.

(13) $${\bar{v}}_{i}=\left(x_{i},y_{i},c_{i}\right)$$

It is, therefore, possible to determine a vector distance between two key points in the skeleton, particularly between two key points that are supposed to be identical. A KPMSE is the sum of distances between key points in TSSCI images that are assumed to have identical values. We calculate the KPMSE as follows:

(14) $$KPMSE=\overset{row}{\underset{r=1}{\sum }}\;\overset{col}{\underset{i=1}{\sum }}\;\overset{ide}{\underset{j=1}{\sum }}{\left(x_{ri}-x_{rj}\right)}^{2}+{\left(y_{ri}-y_{rj}\right)}^{2}+{\left(c_{ri}-c_{rj}\right)}^{2}\;$$

where:

*row*—Number of rows in the TSSCI image;
*col*—Number of columns in the TSSCI image;
*ide*—Number of identical key points in TSSCI.

It is decided to add the KPMSE to the original VAE loss function while using alpha and beta as weights to achieve a balance between the loss function components. Our new loss function, which helps to merge identical key points, is described in the formula below:

(15) Loss=MSE+βDKL+αKPMSE          where 0 ≤ α, β ≤ 1

**Figure 20:**
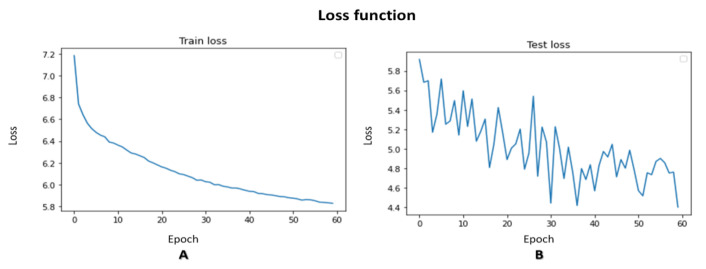
VAE training loss progress ((**A**) train loss, (**B**) test loss).

The VAE training loss progress is illustrated in Figure 20.

**Figure 21:**
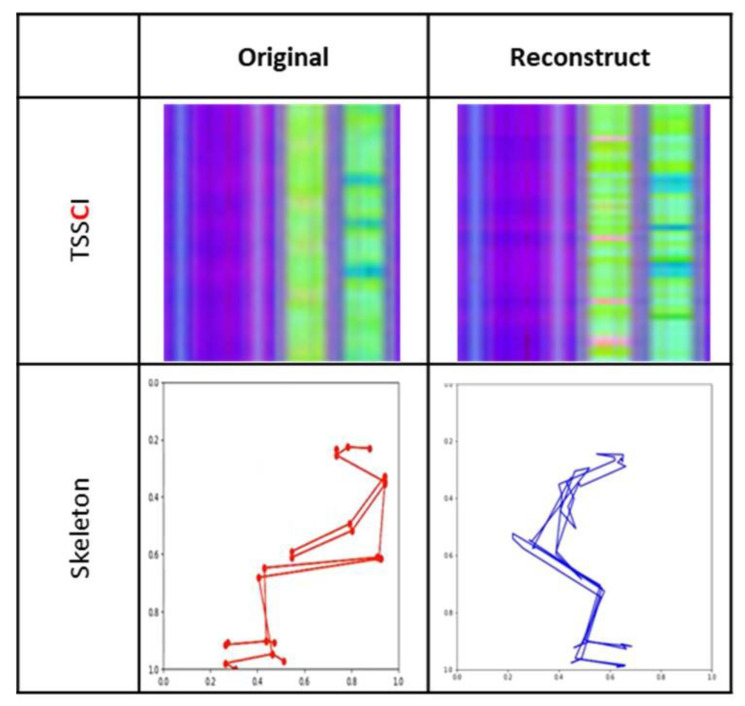
TSSCI encoding and decoding using VAE t-SNE (t-distributed stochastic neighbor embedding) to visualize and explore high-dimensional data.

Figure 21 shows an example of restoring a TSSCI image by compressing until we obtain a latent vector (encoding) and then reconstructing (decoding) the vector back into a TSSCI image.

Our goal is to create synthetic motions similar to the six movements found in our TSSCI picture database, which contains 2004 photos labeled with one of the six movements. We used the TSSCI pictures to train a VAE to recreate each movement. The VAE produces a latent vector for each TSSCI picture, yielding a probabilistic space of latent vectors. We describe the probabilistic space as a cloud of points in an N-dimensional space as a spherical distribution, with a mean of 0 at the center and a standard deviation radius of 1. This spherical distribution encompasses the six motions, and because the TSSCI pictures were successfully separated, we wish to represent the probabilistic space in a two-dimensional graph to demonstrate the apparent distinction between the movements. We used the dimensionality reduction approach t-SNE (t-distributed stochastic neighbor embedding) [29] to visualize and explore high-dimensional data. t-SNE is very good for depicting complicated, non-linear interactions between data points. The t-SNE algorithm maps high-dimensional data points to a lower-dimensional space (usually 2D or 3D) while keeping data point commonalities. The method achieves this by generating a probability distribution over the data points in the high-dimensional space and then mapping the points to the lower-dimensional space while preserving as many similarities as feasible. In more detail, t-SNE starts by computing the pairwise similarities between all the data points in the high-dimensional space (the latent space dimension in our case). We use these similarities to define a probability distribution over the data points, where the probability of a selected point is proportional to the similarity with its neighbors. The algorithm then maps the data points to the lower-dimensional space by optimizing a cost function that measures the difference between the high- and lower-dimensional space probabilities.

**Figure 22:**
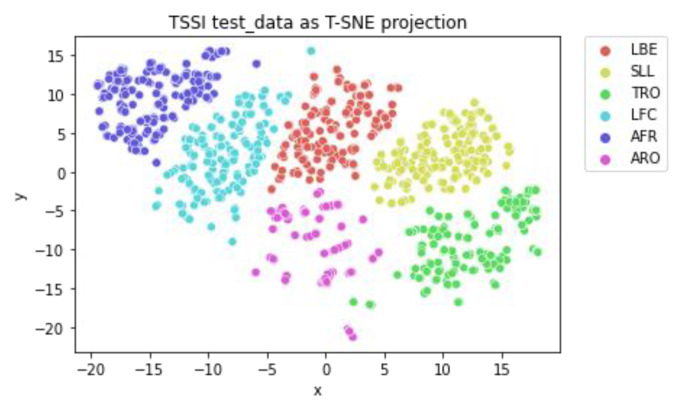
Analyzing the ability to generate similar synthetic movements by using a two-dimensional representation of t-SNE.

As demonstrated in Figure 22, the probability space of the latent vectors representing the characteristics of human movements, or TSSCI image properties, exhibits excellent separation. The separation is centered around the origin, resulting in a symmetrical distribution. This indicates that the VAE has generated a probability space that resembles a normal distribution. Therefore, we can extract a vector from a normal distribution, pass it through the VAE, and obtain a novel movement.

#### 9.3.1. Synthesizes a New Movement by Combining Two Foreign Movements TRO + SLL

**Figure 23:**
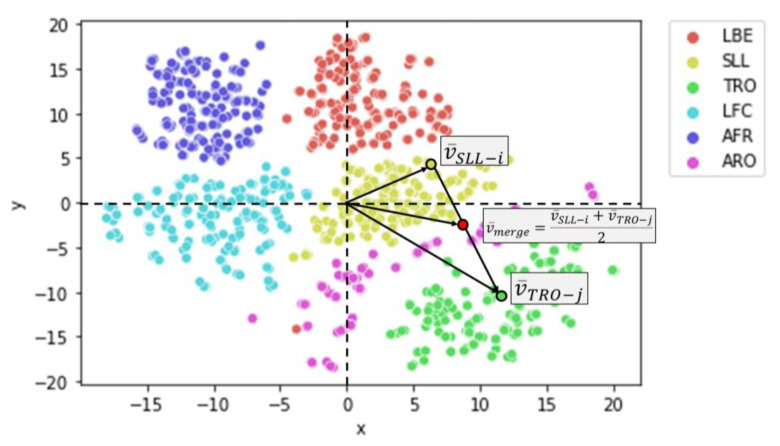
A visual representation of how movement latent vectors merge in the t-SNE dimension.

After employing t-SNE to visualize the distributions of six exercise groups, we seek to showcase the effectiveness of using a super object, specifically TSSCI, to merge two distinct movements into a single, combined motion. Our demonstration involves merging the SLL and TRO movements, which we can characterize by leg and hand-to-chest movement, respectively. Recall that the latent vectors representing the dominant characteristics of each object, in this case, a super object describing a movement, are crucial to our method. Each TSSCI image is transformed into a latent vector using VAE, with each vector serving as a point in an N-dimensional space, where in our example N is 16. VAE converts the distribution of the latent vectors to a normal distribution, meaning that it maps each original latent vector to an equivalent point in a normal distribution. We then choose a latent N-dimensional vector from the normal distribution space belonging to the SLL movement ${\bar{v}}_{SLL-i}$ and one from the TRO movement ${\bar{v}}_{TRO-j}$. We create a vector origin between them (the average of the two vectors) and obtain a new synthetic vector ${\bar{v}}_{merge}$ in the normal distribution space (see Figure 23). This vector represents the combined motion we want to generate. We then inject this vector into the VAE decoder and use it to reconstruct a new movement—the joint movement—which combines the hand and leg movements of the SLL and TRO movements, respectively.

**Figure 24:**
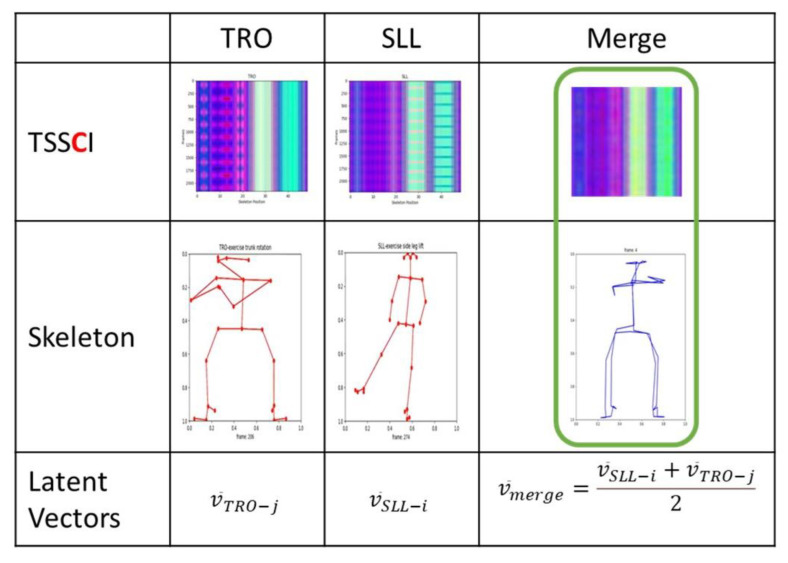
Merging the distinct characteristics of two movements, TRO, and SLL.

Using t-SNE, TSSCI, and VAE allows us to merge the distinct characteristics of two movements and generate a new, unique synthetic movement (see Figure 24).

#### 9.3.2. Generator of Synthetic Movements for Specific Types Using Super Object Method

Our previous demonstrations have showcased the power of the super object method in classifying, compressing, reproducing, and merging movements. We introduce a new capability: a generator that creates a specific type of movement from the six pre-defined movements. The generator operates by converting the latent vector distribution to a normal distribution. Each subgroup of movements has a different mean and variance, and to produce a new movement of a specific type we create a sub-distribution corresponding to the requested subgroup. We then generate a series of latent vectors from each sub-distribution and pass them through the VAE Decoder to obtain a new TSSCI image depicting a sequence of skeletons in certain positions, which, when played in order, produce a new synthetic skeleton movement of the requested type.

**Figure 25:**
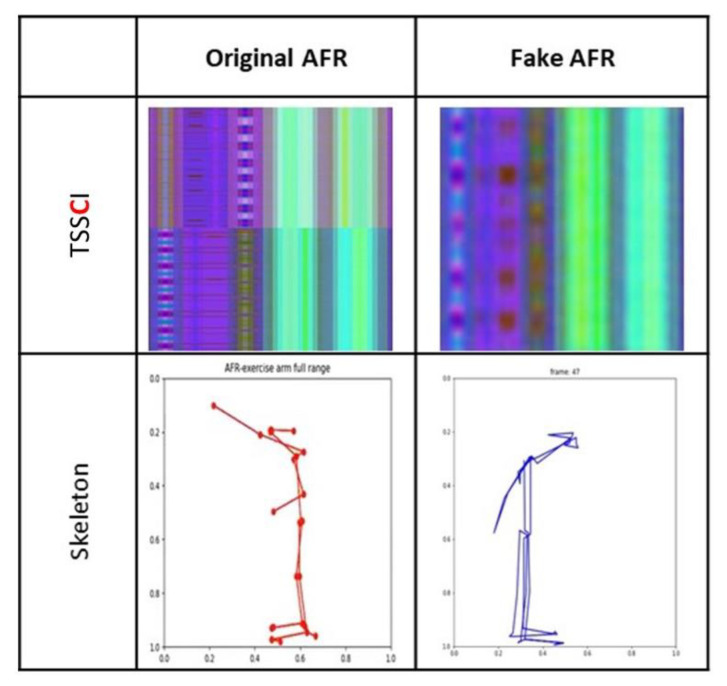
Generator of synthetic AFR movements using super object method.

Figure 25 showcases our ability to generate a new AFR (arm full rotate) movement using the super object method. Notably, the TSSCI image features a dividing line in its center, which reflects the combined performance of the exercise, with the right arm working in the first half and the left arm in the second half. However, our reconstruction only includes the right-arm movement. Hence there is no line in the center. As a reminder, we opted to play the synthetic movement in a mirror image of the original to distinguish between the two. The TSSCI image used to generate the synthetic movement describes a skeleton using 49 points, while the original skeleton uses 25 points. We preserved this format to demonstrate the refinement and authenticity of the synthetic reconstruction using VAE on the TSSCI image. We previously demonstrated how to train the network to generate a skeleton of only 25 points by adding a constraint to the loss function. It provides further evidence of the authenticity of our synthetic movements and their potential applicability in real-world settings.

**Figure 26:**
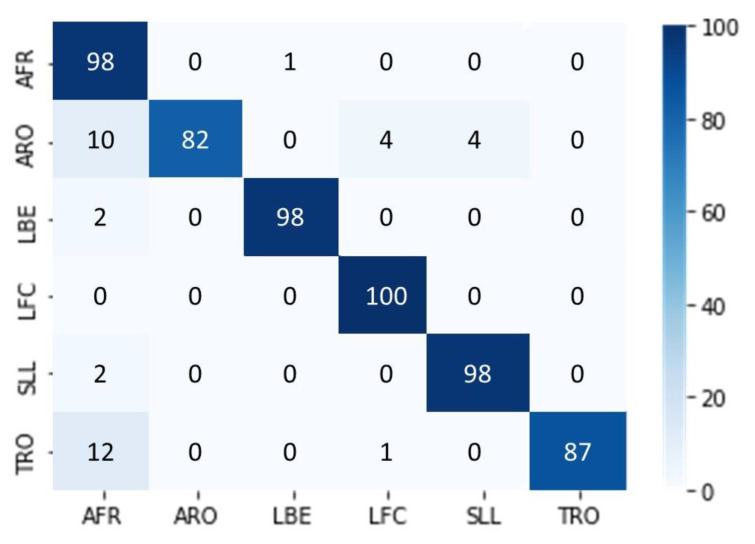
Analyze the quality of creation of 6 movements using a confusion matrix.

We pass the synthetic movements through an EfficientNet-B7 CNN network-based movement classifier to validate the generator effectiveness and present a confusion matrix (see Figure 26). The matrix diagonal clearly illustrates the classifier near-perfect success in classifying all the new fake movements. Overall, this new capability of the super object method allows us to enrich our dataset with more tagged movements of the same type, which is an advanced form of augmentation. Additionally, we can use the movement merging algorithm to create a new set of movements different from the original six.

#### 9.3.3. Utilizing a Siamese Twin Neural Network to Score the Quality of Physical Therapy Exercises Performed by Student Relative to Expert Physical Therapists

In this section, we highlight the effectiveness of the super object via the TSSCI method in providing a score for the quality of exercise performance compared to an expert physiotherapist. Given that our research framework focuses on demonstrating the effectiveness of the super object method, our dataset consists solely of examples from healthy individuals and does not include patients with movement disorders. To validate the effectiveness of our method, we rely on a set of exercises performed by an expert physiotherapist as a reference point. We developed a Siamese twin network based on the EfficientNet network to provide a score for exercise performance. This network features two channels: a reference channel that receives the specialist TSSCI and an identical parallel channel that receives the TSSCIs of all students performing a mix of exercises. Each channel produces a latent vector, and we measure the Euclidean distance between the vectors of the expert and the student performing a particular exercise. The smaller the distance between the vectors, the higher the score, with a score of 100 indicating a perfect mimicked exercise (performing similar movements). Conversely, the greater the distance, the lower the score, with 0 indicating completely different exercise movements.

For scoring we define the variable *f* as follow:

(16) $$f=a\frac{100}{D}$$

where:

*D*—Euclidean distance between the reference and student latent vectors
α—Scaling factor alpha; we empirically chose alpha as 30.

Finally we obtain a score between 0 and 100 by the following formula:

(17) $$s\;=\left\{\begin{array}{c} f\;\;\;\;\;\;\;\;\;\;\;\;\;\;\;\mathrm{if}\;\;\;\;\;\;f<100\\ 100\;\;\;\;\;\;\;\;\;\mathrm{if}\;\;\;\;\;f\geq 100 \end{array}\right.\;$$

where:

s—score, where 0 ≤ s ≤ 100

The Euclidean distance method, commonly used for measuring the similarity between two vectors, suffers from the challenge of an unlimited maximum distance, making it difficult to define a final scale between 0 and 100. To address this challenge, we developed an empirical scoring method that demonstrates the effectiveness of using the super object method rather than focus on providing an accurate score. Our empirical method involves using the inverse of the Euclidean distance and setting a threshold for the maximum value beyond which any similarity score would be considered 100. Specifically, we define a threshold distance of 100, such that any inverse Euclidean distance between the reference and student vectors greater than or equal to 100 is capped at a score of 100. Conversely, any distance less than 100 is multiplied by a scaling factor alpha (in our case, we empirically chose alpha as 30) to obtain a score between 0 and 100. Using this method, we can provide a qualitative score that effectively demonstrates the advantages of the super object method while accommodating the upper unbounded limit challenges of the Euclidean distance method.

**Figure 27:**
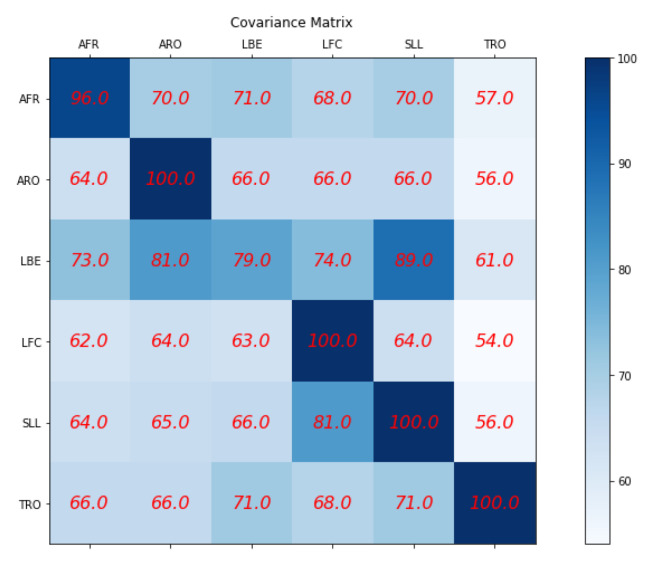
Score of the quality of physical therapy exercises performed by students relative to expert physical therapists.

To validate the effectiveness of our method, we consider an incorrect movement as one that differs from the reference movement performed by the expert physiotherapist. Specifically, we compare the performance of each student movement to the reference movement of the AFR exercise and expect to obtain a high score for those who accurately perform the AFR exercise and a low score for those who perform a different exercise from the expert. To achieve this, we repeat this process six times, using each of the six reference movements performed by the expert as the reference channel. We compare each student movement to the corresponding reference movement and calculate the score using our empirical scoring method. We summarize the scoring results in a confusion matrix, which provides an overview of the accuracy of each type of exercise the students perform (see Figure 27). The matrix rows represent the reference exercise performed by the expert physiotherapist, while the columns represent the type of exercise performed by the students. In each cell, we present the average score obtained for each type of exercise, enabling us to evaluate the effectiveness of our method in accurately classifying different types of movements. The effectiveness of our method is evident from the confusion matrix, which shows that the average score is high for most exercises, except for the LBE exercise. Interestingly, the LBE and the SLL exercises, for which the average score was also lower, share a common characteristic: the dominant movement is related to leg motion. In the LBE exercise, the leg motion is backward, while in the SLL exercise, it is sideways, which explains the system confusion during the scoring process. To address this limitation, we can continue the training process or explore alternative methods, such as using the EfficientNet network as a whole classifier to output a vector of probabilities for each student exercise. We can use this vector of probabilities as a score for the movement or calculate the Euclidean distance between the probability vectors of the expert and the student. However, since our study aims to demonstrate the effectiveness of the super object method for human movement classification, we believe that our empirical scoring method is sufficient to prove the effectiveness of our approach.

#### 9.3.4. Comparing the Quality of Synthetic Exercise Generated by VAE to That Provided by Experts Using a Siamese Twin Neural Network

We repeat the scoring process to complete our evaluation using the synthetic exercises we created with VAE. It enables us to demonstrate the quality of the synthetic movements and the effectiveness of our scoring method based on Siamese twin networks. Siamese twin networks have proven efficient for measuring the similarity between images and objects in various contexts. We aim to illustrate that any existing algorithm designed for analyzing the content of an image, particularly object recognition, can be used for analyzing movements based on the super object method. It shows the versatility and applicability of our approach beyond the scope of movement classification.

**Figure 28:**
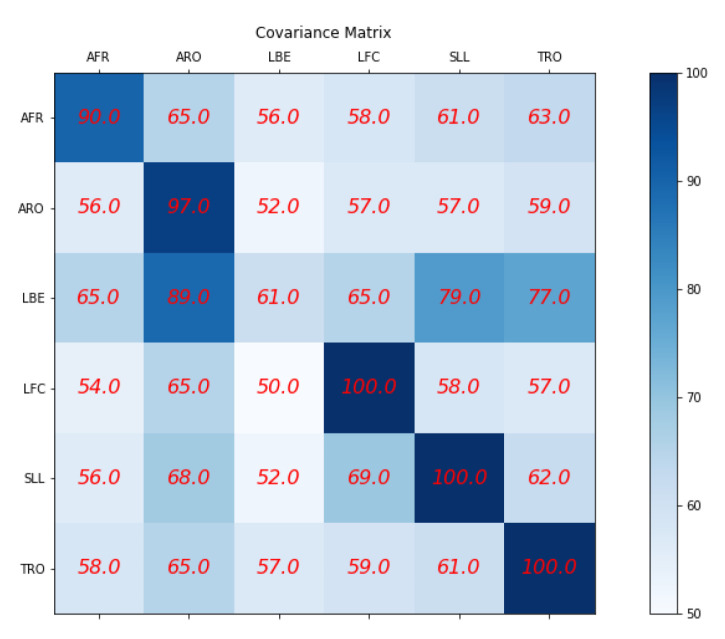
Comparing the quality of synthetic exercise generated by VAE to that provided by experts.

The effectiveness of our method is evident from the confusion matrix presented in Figure 28, which shows that the average score is high for most exercises in the diagonal as expected.

Please note that the majority of the outcomes detailed in this chapter were achieved using Python code, which is available in the Supplementary Materials section of this article.

## 10. Discussion

Our method enables the representation of a series of human movements as one object by converting multi-person key point detection algorithms to RGB-colored TSSCI images. X, Y, and confidence level—C coordinates are used to represent each of the 25 key points in the skeleton. All skeletons are grouped together in three dimensions (3D arrays—x, y, and confidence level c), obtained by multiplying the number of frames by the number of key points. In order to represent the array in RGB, it is converted into the red color channel, which represents the X coordinates, the green color channel, which represents the Y coordinates, and the blue color channel, which represents the C coordinates. Thus, the TSSCI represents the composition of the exercise from beginning to end as an abstracted color image. This new approach allows us to consider a human exercise as an object within a TSSCI image. As an example, in a typical image containing a cat object, a CNN network trained to tag cats will successfully tag most of the cats in a set of images, regardless of where they are within the image, how large they are, and whether they are rotated. In most cases, the video clip of the therapist will contain the entire exercise from the beginning to the end. As a result, the exercise (which is our super object) takes the entire TSSCI (every raw in the image belongs to this exercise), as if the image contained a large cat. In order to prepare for and repeat the exercise, patients normally prepare themselves prior to and after the actual exercise. The TSSCI image reflects this as an object (exercise) situated in the image center, whereas the top and bottom of the image are irrelevant (similar to a background in a typical image). As a result of this mode, the cat appears small and in the center of the picture, while the rest of the image contains mostly background, as if it were taken from a distance. CNNs extract feature vectors (latent vectors) that are representative of the properties of the object being classified, while filtering out other objects and backgrounds and disregarding the dimensions or orientation of the object. Using this new approach of treating the human motion as an object in a TSSCI image, we are able to use a CNN network to extract the latent vector of the human motion. By extracting a latent vector, which distills the characteristics of a movement into a one-dimensional vector, a variety of actions can be accomplished. The latent vector can be injected into a fully connected network, allowing us to classify and label movements. Another option is to inject the latent vector into a Siamese twin network, allowing us to compare therapist and patient and score the patient exercise relative to the therapist. We can create a probabilistic space of various latent vectors by using a CNN network with many patients who try to mimic the same exercise. Keeping in mind that a vector is by definition a point in an N-dimensional space, our latent vector is also a point in an N-dimensional space. As a result, creating multiple latent vectors from many patients produces a cloud of points in the N-dimensional space that represents our probability space of a specific exercise. While the number of points in the probability space for this specific movement is infinite, we have created only a finite collection of points. These points are referred to as “existence points” or “existence,” whereas the probability space between the existing points is referred to as “nothing.” Using the VAE network, we are able to add synthetically virtual existence points to the “nothing” based on the “existence” points. The creation of the new virtual points allows us to create a completely new fake TSSCI image from each new point utilizing VAE generator, thus enriching the number of points in our probabilistic space for this particular exercise. Therefore, we can generate from the faked TSSCI image a video showing the movement of skeletons, describing the movement performed by a fake person, which will increase our dataset for training other neural networks able to perform more complex tasks.

### 10.1. Dataset

We divided our dataset into three parts. The division of it into three parts provides a comprehensive understanding of the different types of environments. The exercises performed by students from the University of Prague under laboratory conditions provide a controlled environment for evaluating the performance of the exercises. It allows for a clear understanding of the effect of the exercises on the students and provides a sterile environment baseline for comparison. On the other hand, the exercises performed by students from Ben Gurion University in an uncontrolled environment provide a real-world scenario of how patients may perform the exercises in the home environment. It provides valuable insights into the practicality and effectiveness of the exercises in a more natural setting. Finally, the exercises the expert physiotherapist performs under controlled laboratory conditions serve as the ground truth, allowing for comparing and measuring the other exercises performed. It provides a benchmark for evaluating the performance of the exercises and allows for a thorough understanding of the efficacy of the exercises. Dividing the dataset into these three parts provides a comprehensive and well-rounded understanding of the exercises and their performance in different scenarios. We felt it appropriate to expand a little beyond the scope of the discussion of this paper and point out that “existence,” “nonexistence,” or “nothing” are central philosophical themes. According to Plato, non-physical ideas, or Forms (“nothing”), are more real than the physical world we perceive (“existence”). According to him, physical objects (in our case, actual movements) are imperfect copies of these non-physical Forms (movements performed by a fake person or merely fake movements), and that the Forms are eternal and unchanging. This technique of creating virtual points within the space of “nothing” by using the points of “existence” could be called “computational imagination” or “ computational creativity”.

### 10.2. Efficient TSSCI Processing with Negligible Resource Consumption

The low resource consumption of TSSCI is a significant advantage that can have a notable impact on human movements analysis. The technique efficiently combines a series of frames into one image, which can be also processed by programs such as OpenPose and MediaPipe that extract the skeletons from the video. These programs typically process 30 frames per second. An average human exercise consists of thousands of frames, say 1000, for this discussion. From the tests we conducted, the TSSCI image processing time using the EfficientNet network is approximately 20 milliseconds. In comparison with a video frame, which has at least 512 × 512 pixels, TSSCI has a small image size of 49 × 49 pixels. Additionally, the abstract structure and image orientation, primarily in the y direction, can enhance network convergence. Assuming the time required to extract a skeleton from a frame is T, according to our measurements the TSSCI calculation time is only a quarter of T. We need to process only one TSSCI per exercise. Consequently, for an exercise, it would take 1000T to extract all the skeletons and only a quarter of T to process TSSCI with a neural network. It is worth mentioning that the creation of TSSCI is insignificant as it only aggregates skeletal data into a single array. In summary, using TSSCI as a super object approach has a negligible impact on the total processing time, accounting for only 0.025% of the total processing time compared to the time required to extract the exercise skeletons. It highlights the efficiency and potential of TSSCI to enhance human body movement analysis while minimizing resource consumption. Our results are promising and have implications for various sectors, such as healthcare and fitness, where efficient video analysis can make a significant difference.

## 11. Conclusions

In this work, we presented a novel and versatile approach to reference human movements, based on super object in the form of a TSSCI image. Our solution provides a generic method for analyzing and processing human movements, using standard deep learning network architectures, allowing for a variety of tasks such as classification, measurement, prediction, completion, and improvement of movements. To demonstrate the effectiveness of our method, we utilized the open-source code from OpenPose [8] and MediaPipe [9], generously provided by the respective authors. This code enabled us to extract a human skeleton graph from a video and consolidate the results into a TSSCI image. We focused on measuring physiotherapy exercises using a dataset of approximately 100 students who performed six physical therapy exercises prescribed by an expert physiotherapist. The data set was divided into three types: exercises performed by an expert, exercises performed in a controlled environment and under laboratory conditions, and a third group who performed the exercises freestyle at home. We demonstrated the versatility of our approach by injecting identical TSSCI images into three different networks (EfficientNet, variational auto-encoder, and Siamese twin networks) for performing different tasks. We successfully classified human movements performed under laboratory conditions and with high accuracy for movements performed under uncontrolled, noisy conditions, except for cases where there were not enough examples for training. Furthermore, we demonstrated the use of a VAE architecture to generate new synthetic skeletons and even merged different movements to create a unique synthetic movement. We explained the VAE architecture using the t-SNE algorithm to present the N-dimensional distribution space of the latent vectors in two dimensions. Finally, we presented the use of the TSSCI method in assessing the performance of physical therapy exercises compared to a specialist physiotherapist using a Siamese twin network. We proposed an empirical scoring method for the quality of the exercise performance and summarized the results in two confusion matrices. Our experiments demonstrate the effectiveness of our super object method in human movement analysis. In conclusion, the combination of convolutional neural networks (CNNs), OpenPose, and MediaPipe can be effectively utilized to evaluate physical therapy exercises performed by patients in a home setting relative to the performance of a remote therapist. The study demonstrates the potential of computer vision techniques in physical therapy and the significance of precise and timely evaluations in enhancing patient results. The super object method was shown to be a practical approach to analyzing human movements, and the Siamese twin network based on the EfficientNet network provided a score for exercise performance. The results of the experiment provide evidence of the method viability and effectiveness, as demonstrated by the confusion matrix. The study can be used as a basis for further research and development in computer vision and physical therapy. Careful consideration of resolution, orientation, and camera stability is essential to ensure that model predictions are accurate and reliable.

## Figures and Tables

**Table 1:** TSSCI Applications.

Neural Network	Regular Image	TSSCI Application
EfficientNet-B7	Object classifier	A system for classifying and labeling movements
UNET	Semantic segmentation	Colors and marks the super object inside the TSSCI image. Allows extrapolation between two movements.
YOLO	Object detection and tracking	Locating a particular movement in a film. Includes the option to extract specific movements.
ESRGAN	Super-resolution	Enhancing motion captured at a low frame rate to a higher frame rate.
DAE or DnCNN	Denoising auto-encoder or a denoising convolutional neural network	Restoration of the skeleton’s missing key points
NST	Transfer the style of one image to the content of another image	Changing dance style from hip-hop to ballet while maintaining the original movements
DC-GAN	Generating fake images	Creating fake movements
VAE-based Image Composition	Generate new images that combine features from the two images	Combining two different movements, such as jumping and clapping, to create a new movement
Transformer-XH	Predict the next frame in a video	Predict the next movement in a sport game
Grid-CNN	Predict a 3D model from 2D images (stereo reconstruction)	Create a 3D model of the skeleton from a 2D skeleton (stereo reconstruction)
DALL-E	Generate images from natural language descriptions	Create an all-encompassing choreography based on natural language descriptions

## Data Availability

Link to publicly archived datasets analyzed and generated during the study: https://bit.ly/bgu_anonymous_dataset, accessed on 6 March 2023.

## Supplementary Materials

The following supporting python code examples and some general explanations can be downloaded from: https://github.com/yoramse/TSSCI.git, https://bit.ly/bgu_pyhton_code_example and from https://bit.ly/BGU_Extra_Info, (accessed on 6 March 2023).
